# Personal

**Published:** 1902-05

**Authors:** 


### PERSONAL.

DR. NELSON H. HENRY, adjutant-general of the New York National Guard, visited Buffalo recently at which time he made an inspection of the 65th regiment, which is under command of Brevet Brigadier-general S. M. Welch. After the review, General Henry and several other officers of the guard were entertained by General Welch at the Saturn Club.

---

DR. L. S. MCMURTRY, of Louisville, read a paper before the Buffalo Academy of Medicine, section on obstetrics and gynecology, Tuesday evening, April 23, 1902, the title of which was The prophylaxis of uterine cancer, clinically considered. The subject was admirably presented and the meeting was highly enjoyed by a large gathering of the Fellows.

---

Dr. Charles G. Stockton, of Buffalo, who went to Europe in February, and with Mrs. Stockton took the Mediterranean tour, has returned and resumed his professional work.

---

Dr. Edward J. Meyer, of Buffalo, who has been in Florida for several weeks has recovered from his illness, and has returned and resumed his practice.

---

Dr. A. E. Persons has returned to Buffalo to resume the practice of his profession, and has established his offices at 19 West Tupper Street.

---

Dr. Roswell Park, of Buffalo, will deliver the doctorate address at the commencement of Yale Medical College, June 24, 1902.

---

Dr. G. F. Mills, of the Manhattan State Hospital, New York, has been transferred to the staff of the Buffalo State Hospital.

---

Dr. Nelson G. Russell, of Buffalo, has removed from 191 Allen Street to 448 Franklin Street.

---

Dr. C. G. Leo-Wolf, of Niagara Falls, has been appointed health officer of the cataract city.

---

Dr. John H. Pryor, of Buffalo, has removed from 56 Allen Street to 475 Franklin Street.

---

Dr. John T. Harris, of Tonawanda, has been appointed health physician of that city.

